# Personalized Cell Therapy for Patients with Peripheral Arterial Diseases in the Context of Genetic Alterations: Artificial Intelligence-Based Responder and Non-Responder Prediction

**DOI:** 10.3390/cells10123266

**Published:** 2021-11-23

**Authors:** Amankeldi A. Salybekov, Markus Wolfien, Shuzo Kobayashi, Gustav Steinhoff, Takayuki Asahara

**Affiliations:** 1 Kidney Disease and Transplant Center, Shonan Kamakura General Hospital, 1-1370 Okamoto, Kamakura 2478533, Japan; shuzo@shonankamakura.or.jp; 2 Shonan Research Institute of Innovative Medicine, Shonan Kamakura General Hospital, 1-1370 Okamoto, Kamakura 2478533, Japan; 3 Department of Systems Biology and Bioinformatics, University of Rostock, Ulmenstrasse 69, 18057 Rostock, Germany; markus.wolfien@uni-rostock.de; 4 Department of Cardiac Surgery, Rostock University Medical Center, 18059 Rostock, Germany; gustav.steinhoff@med.uni-rostock.de; 5 Department Life, Light & Matter, University of Rostock, 18057 Rostock, Germany

**Keywords:** cell therapy, chronic limb-threating ischemia, peripheral artery disease, diabetes, atherosclerosis obliterans, thromboangiitis obliterans, personalized medicine, artificial intelligence, machine learning, genome-wide association studies, transcriptome-wide association studies, clonal hematopoiesis of indeterminate potential

## Abstract

Stem/progenitor cell transplantation is a potential novel therapeutic strategy to induce angiogenesis in ischemic tissue, which can prevent major amputation in patients with advanced peripheral artery disease (PAD). Thus, clinicians can use cell therapies worldwide to treat PAD. However, some cell therapy studies did not report beneficial outcomes. Clinical researchers have suggested that classical risk factors and comorbidities may adversely affect the efficacy of cell therapy. Some studies have indicated that the response to stem cell therapy varies among patients, even in those harboring limited risk factors. This suggests the role of undetermined risk factors, including genetic alterations, somatic mutations, and clonal hematopoiesis. Personalized stem cell-based therapy can be developed by analyzing individual risk factors. These approaches must consider several clinical biomarkers and perform studies (such as genome-wide association studies (GWAS)) on disease-related genetic traits and integrate the findings with those of transcriptome-wide association studies (TWAS) and whole-genome sequencing in PAD. Additional unbiased analyses with state-of-the-art computational methods, such as machine learning-based patient stratification, are suited for predictions in clinical investigations. The integration of these complex approaches into a unified analysis procedure for the identification of responders and non-responders before stem cell therapy, which can decrease treatment expenditure, is a major challenge for increasing the efficacy of therapies.

## 1. Introduction

Peripheral arterial disease (PAD) is the second leading cause of mortality and morbidity among cardiovascular diseases (CVDs) [1,2,3]. According to the global disease-burden data, the incidence of PAD in low-income and middle-income countries increased by 28.7%, while that in high-income countries increased by 13.1% compared to the preceding decade [1,3]. Chronic limb-threatening ischemia (CLTI) is clinically defined as chronic and severe limb-perfusion insufficiency that leads to tissue ulceration and gangrene [2]. The poor prognostic outcomes and high mortality rate of PAD are attributed to the following three main “conventional” risk factors: lifestyle (lifestyle risk factors subcategory, such as western diet, cigarette smoking, sedentary lifestyle, and alcohol consumption); comorbidities (comorbidities risk factors subcategories, such as hypertension, dyslipidemia (including all atherogenic lipid subsets or familial hypercholesterolemia), diabetes mellitus, obesity, homocysteine, high-sensitive C-reactive protein (hsCRP) and fibrinogen, and chronic kidney disease; and the genetic background risk factors subcategory, such as race and ethnicity [1,2,3]. Conventional cardiovascular risk factors contribute to the development of PAD. In the presence of four of the subcategory risk factors, the incidence rate of PAD can increase up to 140 cases per 100,000 individuals [4]. Clinical evidence suggests that additional or unidentified genetic risk factors may contribute to CVD development [5,6]. For example, the accumulation of somatic mutations in hematopoietic cells throughout life, which is referred to as clonal hematopoiesis of indeterminate potential (CHIP) (see Box 1), contributes to the development of CVD [5]. Several studies have demonstrated that the frequency of *JAK2 V617F* mutations in patients with atherosclerosis obliterans (ASO) of the lower extremities is five-fold higher than that in healthy controls [7]. Moreover, CHIP carriers are associated with a significantly higher risk of CVD events than non-carriers, such as atherosclerosis, PAD, and myocardial infarction [5,7]. Recent studies have demonstrated that 20% of the elderly population (60 to 90 years old) harbors mutated *DNMT3A*, *TET2*, *ASXL1*, *JAK2*, and *NOTCH1* in hematopoietic stem cells (HSCs), such as CD34^+^ and CD133^+^ cells. CD133^+^ cells have applications in advanced treatment for patients with terminal-stage PAD [5,8]. Thus, there is a need to reassess the stem/progenitor cell genetic background to develop improved therapeutic strategies for PAD [9,10].

Box 1 **Definition of terms.**

Clonal hematopoiesis is the expansion of a clonal population of hematopoietic cells with one or more somatic mutations [6,11].

CHIP is defined by the absence of definitive morphological evidence of hematological neoplasms and the presence of a somatic mutation with a variant allele frequency of at least 2% to 4% [11].

Somatic mutations occur in somatic cells and cannot be inherited (only tissues derived from the mutated cell are affected) [6,12].

Germline mutations occur in gametes and can be passed on to offspring (every cell in the entire organism will be affected).

Variant allele frequency (VAF) is the percentage of sequence reads matching a specific DNA variant divided by the overall coverage at that locus [13].

Therapeutic angiogenesis with stem/progenitor cells is a promising strategy for treating ischemic tissue and preventing major amputation. Stem cell application accelerates angiogenesis (formation of new vessels from pre-existing vessels), vasculogenesis, and de novo synthesis of new vessels from transplanted and circulating precursor cells [14]. Previously, intra-arterial or intramuscular transplantation of stem cells has been performed in PAD patients with Rutherford grades 3–6. Some studies have obtained promising results using this strategy, including improved limb-blood perfusion, increased major amputation-free periods, and enhanced quality of life (Table 1). However, the response rate for stem cell-based therapy in patients with PAD exhibiting comorbidities (such as diabetes, hypertension, and dyslipidemia) and with risk factors (such as cigarette smoking) is lower or poorer than that in placebo-treated groups (Table 2). Hence, patients who respond or do not respond to stem/progenitor cell therapy must be determined before starting a clinical trial in cases of advanced CLTI. Previously, we determined cases responding to stem/progenitor cell therapy among patients with heart failure [15].

Personalized stem cell-based therapy approaches employing several clinical biomarkers, disease-related genetic-trait evaluation methods, such as the analysis of the findings of transcriptome-wide association studies (TWAS)/genome-wide association studies (GWAS)) of PAD, and advanced analyses with state-of-the-art computational methods (such as machine learning- (ML) based prediction) can contribute to clinical investigations [10]. The integration of these complex approaches is a major challenge for increasing the efficacy of therapies and decreasing treatment costs. Here, the pros and cons of the transplantation of granulocyte colony-stimulating factor- (G-CSF) mobilized CD34^+^ or endothelial progenitor cells (EPCs) were compared with those of bone marrow-derived mononuclear cells (BMMCs) and peripheral blood-derived mononuclear cells (PBMCs) by analyzing the results of randomized, placebo-controlled clinical trials with a focus on clonal hematopoiesis (CH). Additionally, the possibility of increasing the efficacy and safety of stem cell transplantation for different cell types based on somatic mutations in HSCs and studies on predictors that can identify the responder (R) and non-responder (NR) groups before stem cell therapy have been highlighted.

## 2. Personalized Stem Cell Therapy for Patients with PAD Based on Phenotype and Genotype Findings

Clinical trials of CLTI/PAD have identified several clinical risk factors and biomarkers associated with poor cell transplantation outcomes. Precise evaluation techniques are needed to achieve beneficial clinical outcomes and reduce the financial burden of advanced medical treatment technologies for patients with CLTI/PAD.

### 2.1. Characteristic Features of the R and NR Groups

Various clinical trials of stem/progenitor cell transplantation for PAD or CLTI have identified several predictors and biomarkers of response to cellular therapy (Table 3). Klepanec et al. [25] reported several parameters that delineated the R group from the NR group. Compared with the NR group, the R group exhibited a two-fold higher absolute CD34^+^ cell count but a similar number of total bone marrow mononuclear cells (BMNCs). Additionally, the CRP levels and total leucocyte counts in the R group were lower than those in the NR group. The general inflammation process and the number of CD34^+^ cells are directly associated with the cellular therapy outcomes [25,26]. For example, three independent clinical trials reported that increased levels of IL-6, CRP, serum leucocyte count, fibrinogen, and basic fibroblast growth factor are associated with a weak cellular therapy response (Table 3). Two studies have reported that age < 50 years was an independent predictor of improved cellular therapy response in patients with CLTI [26,27]. Consistently, Jaiswal et al. reported that the frequency of somatic mutations markedly increased in patients aged > 50 years [5,28]. Moreover, patients with myelodysplastic syndrome (MDS) aged > 65 years and harboring *TET2*, *DNMT3A*, and *AXSL1* mutations were susceptible to PAD and systemic inflammation [29]. These findings indicate a correlation between inflammation and CHIP, as well as CVDs, including PAD. PAD and coronary artery disease (CAD) are associated with decreased HSC proliferation and inhibition of angiogenesis, which lead to tissue ischemia and inflammation [15,30]. In the PERFECT trial, ML determined that the response of bone marrow stem cell transplantation-based myocardial regeneration was correlated with *PLCG1*, LPCAT2, *AP1B1*, *AFAP1*, *GRB2*, *KLF8*, and *MARK3* expression levels and serum *EPO* and *VEGF* levels but was not correlated with the expression of CHIP-related genes (*DNMT3a*, *TET2*, and *ASXL1*) (Table 3). Sequencing data revealed that the R group exhibited 161 differentially expressed genes when compared with the NR group. Mutation analysis revealed that the number of specific variants in the R group (48 genes) was lower than that in the NR group (224 genes). Additionally, R-related and NR-related genes determined using ML were correlated with *SH2B3,* as well as with other regulatory genes, such as *NOTCH2, PDCD1/PD-1*, and *CD133* [10].

### 2.2. BMNC Transplantation Alleviates Thromboangiitis Obliterans (TAO), and Purified CD34/CD133^+^ Stem Cell Therapy Alleviates ASO

Clinical studies have revealed that autologous G-CSF-mobilized peripheral blood EPCs exert higher therapeutic effects than BMMCs or PBMCs in patients with TAO (Table 4). Arai et al. [32] demonstrated that the legs injected with CD34^+^ exhibited a significantly higher ankle-brachial index (ABI) (0.56 ± 0.04) than those injected with BMMCs (0.53 ± 0.06) at week 4 after cell therapy. Similar improvements in transcutaneous oxygen pressure (TcPO2) were observed in both CD34^+^-transplanted (from 27 ± 4 to 37 ± 3; *p* < 0.05) and BMT-transplanted groups (from 24 ± 6 to 32 ± 8; *p* < 0.05) at week 4 post-implantation. One clinical trial demonstrated that CD34^+^ cells dose-dependently prevented major or minor amputation when compared with the placebo. At month 6 post-injection, 67%, 43%, and 22% of the control, low-dose-administered, and high-dose-administered groups underwent a major or minor amputation (*p* > 0.137), respectively. This trend continued at month 12 post-injection, with 75%, 45%, and 22% of the control, low-dose-administered, and high-dose-administered groups undergoing amputation (*p* > 0.058), respectively. The amputation rate in the combined cell-treated groups was lower than in the control group (month 6, *p* > 0.125; month 12, *p* > 0.054). The low-dose-administered and high-dose-administered groups exhibited improved amputation-free survival at months 6 and 12 post-injection [24].

The long-term clinical outcomes of CD34^+^ transplantation revealed that compared with those at the baseline, toe-brachial pressure index (TBPI) and T_c_PO_2_ significantly improved at week 12 post-transplantation in both CLI and TAO cases but improved only at week 52 in TAO cases. The improvements in TBPI and T_c_PO_2_ were sustained until weeks 156 and 208, respectively. The ulcers completely healed in all patients with TAO and two patients with atherosclerotic PAD at week 52 [19]. A recent randomized, single-blinded trial evaluated the 12-month treatment outcomes of purified CD34^+^ cell transplantation and PBMC transplantation for advanced TAO. The total amputation rates at month 6 post-transplantation in the CD34^+^-transplanted and PBMC-transplanted groups were 28.0% and 16.0%, respectively (*p* > 0.343), which remained unchanged at month 12 post-transplantation. Furthermore, the complete wound-healing rates did not significantly differ between the groups at months 3, 6, and 12. The two groups exhibited significantly increased ABI, toe-brachial index (TBI), T_c_PO_2_, and pain-free walking time (PFWT) values over time when compared with baseline values (ΔABI, ΔTBI, ΔT_c_PO_2_, and ΔPFWT, respectively) [33]. Another clinical trial evaluated the long-term efficacy of autologous BMMC transplantation in ASO and TAO [34]. The four-year amputation-free rates in the control ASO, BMMC-transplanted ASO, control TAO, and BMMC-transplanted groups were 0%, 48%, 6%, and 95%, respectively. ABI and T_c_PO_2_ significantly increased after one month in the BMMC-transplanted TAO group and remained high during the three-year follow-up visit. In contrast, ABI and T_c_O_2_ significantly increased only in the first month in the BMMC-transplanted ASO group and gradually decreased during the three-year follow-up before finally returning to baseline levels [34]. The 10-year amputation-free survival rates in the autologous BMMC-transplanted and aspirin-treated groups were 85.3% (29/34) and 40% (6/15) (*p* < 0.0019), respectively. Autologous BMMC transplantation significantly decreased the ulcer area (*p* < 0.0001), TBI (*p* < 0.0001), T_c_PO_2_ (*p* < 0.0001), and pain score (*p* < 0.0001) [35]. Moreover, transplantation of autologous stem cells harboring mutations is reported to promote the expansion of mutant blood cell clones, leading to increased risks of CHIP-associated complications [36]. However, patients with TAO are younger than those with ASO. Thus, TAO cases exhibited decreased somatic mutations and inflammation in the peripheral blood (Table 4 and Table 5).

These findings indicate that BMMC or PBMC transplantation is safe and feasible for patients with TAO. The parameters, such as the amputation-free rate (86%–95% in TAO and 40%–50% in ASO) and the enhanced ABI, TBI, T_c_PO_2_, and PFWT values compared with baseline values, indicated the effectiveness of cell therapies. BMMC or PBMC transplantation can exhibit similar efficacy as G-CSF-mobilized CD34^+^ cell transplantation in patients with TAO. However, purified or enriched transplantation of EPCs with a decreased somatic mutation profile must be performed for ASO. Thus, optimal stem cell-based therapy must be determined depending on the type of disease to reduce treatment expenditure.

## 3. CLTI/PAD in Patients with Diabetes Mellitus

**Figure 1:**
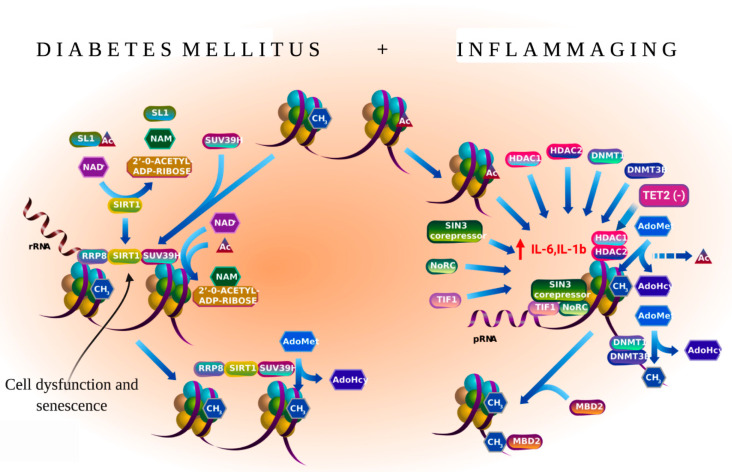
Negative epigenetic regulation of gene expression. The figure was reproduced from Reactome ver. 72 (URL.: https://reactome.org/PathwayBrowser/#/R-HSA-5250941, accessed on 18 January 2021. Stable ID: R-HAS-5250941. Abbreviations: NoRC, nucleolar remodeling complex.

Approximately one third of patients with diabetes mellitus develop PAD, with various grades of disease severity and type [43]. Hence, personalized stem cell therapy can increase the response rate, ameliorate patient-specific clinical parameters, enhance quality of life, and decrease therapy cost. The mobilization of CD34^+^ stem cells is impaired in patients with diabetes mellitus, while patients who previously received G-CSF have a decreased probability of achieving a CD34^+^ stem cell count of >50/μL [46,47]. This may be associated with diabetic bone marrow (BM) autonomic neuropathy, which impairs Lin^−^ cKit^+^ Sca1^+^ cells and EPC mobilization through the upregulation of 66-kDa protein from the *src* homology and collagen homology domains, as well as the downregulation of *sirtulin 1* in mice and humans [46,48] (Figure 1). Teraa et al., reported that CLTI induces alterations in bone marrow vasculature and sympathetic nerve innervation in patients with and without diabetes mellitus [49]. Bonnefond et al. [50] examined the correlation between type two diabetes mellitus (T2DM) and CHIP events in the blood. The occurrence of CHIP events was correlated with T2DM (odds ratio (OR) = 5.3; *p* = 5.1 × 10^−5^), and the correlation was strong when non-obese individuals with T2DM (OR = 5.6; *p* = 4.9 × 10^−5^) were included. Previous studies have revealed that advanced age and a long history of T2DM, atherosclerosis, vessel formation, and revascularization are associated with persistent glucotoxicity, oxidative, and inflammatory damages, which may impair vascular regenerative cell lineages [48,51].

Moreover, chronic inflammation and enhanced reactive oxygen species (ROS) production can lead to dysfunctional HSC self-renewal in the endosteal niche, which leads to persistent premature mobilization of HSCs into the peripheral vessels and, consequently, the exhaustion of the reservoir of early myeloid progenitor cells with proangiogenic secretory function [52,53]. This can be attributed to the genetic background or CH events in T2DM, which impair vascular regeneration and promote inflammation through the above-mentioned mechanisms. For example, clonal mosaic event carriers (71.4%) with T2DM exhibited a higher prevalence of vascular complications, such as microvascular and macrovascular lesions, than non-carriers with T2DM (37.1%) *p* = 7.7 × 10^−4^) [50]. This indicates that multiple risk factors contribute to T2DM-related stem/progenitor cell-quality impairments, including somatic mutations (Figure 1).

Diabetes affects the BM niche, decreases the availability of circulating stem/progenitor cells, impairs the function of vascular progenitors, and prevents mobilization. Hence, ex vivo incubation of BM-derived stem/progenitor cells of diabetic animals with growth factor cocktails can restore vasculogenic and anti-inflammatory potential in vitro and in vivo [54,55]. Furthermore, enriched cell transplantation in acute myocardial ischemia [56,57] and diabetic animal wounding models [48,55] effectively restored the vasculogenic and anti-inflammatory potential in a small number of regeneration-associated cells, such as EPCs and T regulatory cells, and alternatively activated macrophages. This may indicate that “exhausted” or dysfunctional cells in diabetes mellitus gain several regenerative functions after incubation with growth factor cocktails.

In summary, genetic alterations are high in patients with diabetes mellitus and PAD. Chronic inflammation caused by increased blood glucose levels and the resulting processes (ROS production and oxidative stress) may impair the function of HSCs and endothelial cell lineages through the introduction of additional mutations.

## 4. Findings of GWAS on Variations in PAD-Associated Gene Loci Involved in Inflammation, Thrombosis, and Lipid Imbalance

### Findings of GWAS on ASO

**Figure 2:**
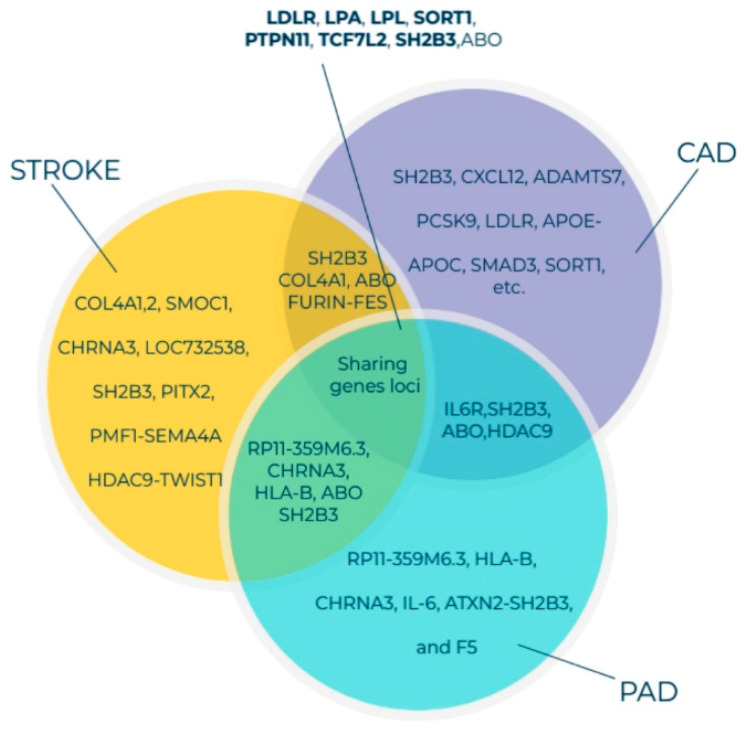
Venn diagram of genes associated with susceptibility to PAD, CAD, and stroke identified through GWAS and the shared gene loci. All listed genes shown here were adapted from the GWAS catalog database for PAD, CAD, and stroke [61,62,63]. Currently, more than 190, 1060, and 327 gene loci have been identified for PAD, CAD, and stroke, respectively. The GWAS 71 catalog accession date is 18 January 2021 Abbreviations: PAD, peripheral arterial disease; CAD, coronary artery disease; GWAS, genome-wide association studies.

High-throughput genotypic platforms, such as hybridization-based single nucleotide polymorphism (SNP) arrays and various next-generation sequencing (NGS) technologies, are widely applied for gene-variant-disease association studies worldwide. In GWAS, the correlation between the phenotypes and the genetic variants across the genome within, around, and between genes is analyzed. Early GWAS on PAD involving a small Japanese population reported that PAD was strongly correlated with rs1902341 or *OSBPL10* gene locus. *OSBPL10* variation may enhance triglyceride levels in patients with dyslipidemia, which indicates that it regulates cellular lipid metabolism [58,59]. Kullo et al. performed a two-stage genomic association study. In the first stage, 537 and 872 SNPs were examined in 1641 PAD cases, as well as 1604 control subjects of European ancestry. Next, the top 48 SNPs associated with PAD were genotyped. The SNP rs653178 in the *ATXN2-SH2B3* locus was significantly associated with PAD in the discovery (OR = 1.23; *p* = 5.59 × 10^−5^), replication (OR = 1.22; 8.9 × 10^−4^), and combined (OR = 1.22; *p* = 6.46 × 10^−7^) cohorts. *SH2B3* encodes an adapter protein that plays a key role in immune- and inflammatory-response pathways, hematopoietic cell regulation in the BM niche, and vascular homeostasis [15,60]. Other GWAS on CVDs (e.g., stroke, PAD, hypertension, and acute coronary syndrome (ACS)) have indicated that the *SH2B3* locus is a key risk factor for the development of CVDs (Figure 2).

**Figure 3:**
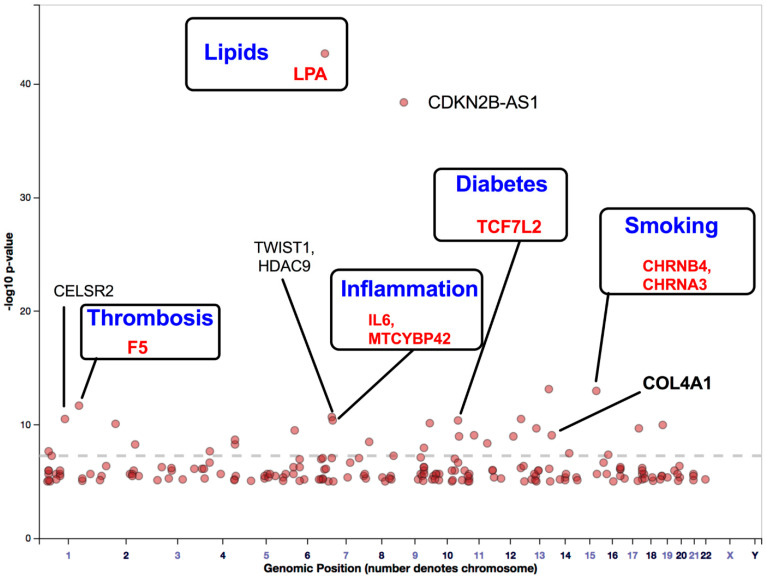
Gene loci associated with PAD identified through GWAS. This figure was modified from the GWAS catalog database with 261,600 patients with PAD. The seven GWAS have been registered as PAD traits (study accession # GCST000720, GCST002504, GCST003154, GCST009133, GCST009134, and GCST008474 downloaded on 18 January 2021). Here, we show a possible improvement of five genes that may reduce disease-development risk and enhance stem cell therapy efficacy. Abbreviations: PAD, peripheral arterial disease; GWAS, genome-wide association studies.

Previously, we had reported that *SH2B3/LNK* mutations in the NR group were higher than those in the R group and that altered *SH2B3/LNK* expression, along with regeneration-associated pathways, contributes to cardiac healing [13,15]. One Asian population-based GWAS identified the following three novel PAD susceptibility loci with genome-wide significance: *IPO5/RAP2A* (a member of the importin beta family that promotes apolipoprotein A-1 excretion; *p* = 6.8 × 10^−14^), *EDNRA* (*p* = 5.3 × 10^−9^), and *HDAC9* (*p* = 8.8 × 10^−8^). For example, the *HDAC9* locus was identified in GWAS on stroke, ACS, and PAD [64,65]. *HDAC9* increases the risk of disease development by enhancing the atherosclerosis process in large- and small-caliber vessels [64,65]. GWAS involving Hispanic/Latino ethnic subgroups have demonstrated ethnic diversity of the PAD-related gene locus among the European and Asian populations. Two genome-wide significant associations were identified between ABI and the SNP rs4466200 at the *COMMD10* locus in the Puerto Rican population and the SNP rs12520838 at the *SYBU* locus in the Caribbean population, which suggested ethnic genetic diversity of PAD on different continents [66]. Klarin et al. [62] recently identified 19 novel PAD loci, among which 18 were not reported previously (Figure 3). A large cohort study included 31,307 PAD cases and 211,753 controls and examined approximately 32 million DNA sequence variants in veterans of European, African, and Hispanic ancestry. A phenome-wide association study was applied to examine the association of risk variants across various phenotypes, which detected 11 PAD risk variants. A significant association was demonstrated between all three (coronary, cerebral, and peripheral) vascular beds, lipids (*LDLR*, *LPA*, *LPL*, and *SORT1*), hypertension (*PTPN11*), and diabetes (*TCF7L2*). Variants in the *RP11-359M6.3, HLA-B, CHRNA3*, and *F5* loci were uniquely associated with PAD. This indicated that a tobacco-smoking-related gene (*CHRNA3*) and a thrombosis-related gene (*F5*) might play an essential role in PAD but not in other arterial diseases (Figure 3). Cyclin-dependent kinase inhibitor 2 B antisense (*CDKN2B-AS1*), which enhances lipid uptake and accumulation, is another risk variant that is upregulated in atherosclerotic plaque tissues and human primary macrophage-derived foam cells. Long, non-coding RNA *CDKN2B-AS1* recruits EZH2 and CTCF in the *CDKN2B* promoter region and consequently inhibits *CDKN2B* transcription by promoting histone methylation [67].

GWAS have identified several gene loci that significantly contribute to PAD development. Most of the identified gene loci share similar traits with CADs and stroke (Figure 2 and Figure 3). Several genes are directly associated with traditional PAD risk factors (e.g., hyperlipidemia, hypertension, aging, and diabetes). These classical risk factors impair stem/progenitor cell-dependent therapeutic effects (Figure 3). Hence, loci identified using GWAS were integrated into the findings of TWAS to detect gene-trait associations. TWAS leverage expression reference panels (expression quantitative trait loci (eQTL) cohorts with expression and genotype data) to identify gene-trait associations from GWAS datasets [68]. This methodology may help to understand transcription loss in the tissues or cells during PAD development. Moreover, high-throughput deep-sequencing methods enable the discovery of somatic mutations and epigenetic regulation of genes, proving valuable insights into single-nucleotide variants, SNPs, and mutation load that are not currently available in GWAS.

## 5. Genetic Alterations in PAD

### 5.1. Somatic Mutation in PAD

In 2019, 240 million cases of PAD were reported worldwide. The prevalence of PAD markedly increases with age (from 6% in individuals aged 40 years to approximately 27% in individuals at an advanced age [42] https://www.cdc.gov/heartdisease/PAD.htm. Accessed on 18 October 2021) These statistical data are positively correlated with the CHIP prevalence in patients with CVD. CHIP rarely occurs in the young population. However, the prevalence of CHIP increases to 10%–20% in the aging population [5] (see Table 5). 

Muendlein et al. revealed that the prevalence of *JAK2 V617F* mutation frequency in a cohort of 287 patients with sonographically confirmed PAD was higher than that in 997 healthy subjects. The acquired *JAK2 V617F* mutation frequency in patients with PAD was five-fold higher than that in healthy individuals (*p* < 0.001). Interestingly, the frequency of mutations in patients with PAD markedly decreased in patients who received aspirin (*p* < 0.003) [7]. Moreover, *JAK2* mutations are reported to play an essential role in systemic inflammation, coagulation, decreased proliferation and angiogenesis, and thrombosis through the activation of the downstream STAT1,6, MAPK, and PI3K/AKT signaling pathways [73]. Mechanistically, patients with *JAK2 V617F* mutation exhibit decreased blood endothelial-cell outgrowth and enhanced expression of interferon-related genes, including serine protease inhibitor B2, early growth response protein 1, and chemokine ligand 2 [30]. This indicated that individuals with *JAK2 V617F* mutation exhibit sustained inflammation/permeability, limited cell growth (or angiogenesis), and EPC senescence when compared with healthy controls [30]. Walsh et al. [74] recently demonstrated that *JAK2 V617F*-mediated CH promotes pathological cardiac remodeling by enhancing the pro-inflammatory properties of circulating myeloid cells, such as secretion of IL-1b, IL-6, and TNFα. Clinical trials on previously transplanted CD34^+^ or cultured “early EPC” and (modestly) “late EPC” did not reveal beneficial effects on ischemic diseases (Table 4) [24,75]. This may suggest that the cell product is impaired due to CHIP. Additionally, a comparative study of somatic mutation profiling of CD34^+^, HSCs, and circulating endothelial cells (CECs) revealed that in 70% of patients with MDS—both CECs and HSCs—harbor the most frequently mutated genes, such as *JAK2, ASXL1, TET2, NOTCH1*, and *SRSF2* [76].

### 5.2. Inflammaging and PAD

**Figure 4:**
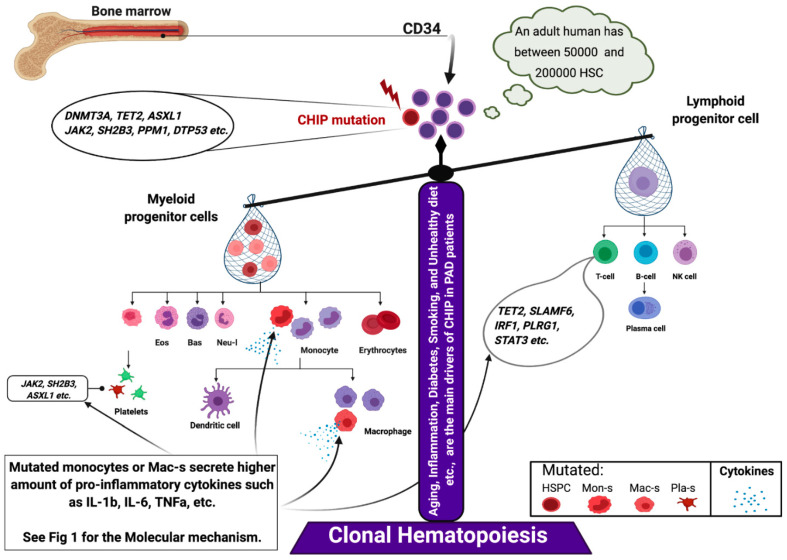
CHIP-induced hematopoiesis. Abbreviation: CHIP, clonal hematopoiesis of indeterminate potential.

Inflammation promotes several pathologies associated with aging, which is called “inflammaging” [77]. Recent studies have reported the downstream regulation of mutated genes involved in the inflammatory process. For example, *TET2* deficiency in hematopoietic cells promotes an aberrant production of inflammatory cytokines/chemokines, such as IL-6 and IL-1b in macrophages. This provides a direct link between *TET2* loss and microenvironmental changes within the BM niche [78,79] (Figure 4). Moreover, a recent large cross-sectional analysis of (*n* = 750, 249) patients with MDS across the United States revealed that patients with *TET2*, *DNMT3A*, and *AXSL1* mutations were associated with a high prevalence of PAD (prevalence among individuals aged <65 and >65 years was 14.5% and 43.2%, respectively; *p* > 0.0001) and CAD (prevalence among individuals aged <65 and >65 years was 9.1% and 17.3%, respectively; *p* < 0.04) [29]. These findings indicate that CHIP carrier patients with PAD aged >65 were at three- to four-fold higher risk of developing PAD than non-carriers. Patients with PAD who have previously undergone transplantation with cells, such as BMNCs or PBMCs or with enriched CD34 cells may exhibit a pro-inflammatory cell phenotype due to somatic mutations in these cells. Transplantation of enriched CD34 cells exacerbates cell therapy outcomes rather than regenerating ischemic tissues [10,15,80]. Transplanted mutant clones increase clone size after auto-transplantation of cells and enhance the secretion of inflammatory cytokines and CRP (Figure 4) [36,81]. The enhanced levels of pro-inflammatory cytokines maintain mutant hematopoietic stem/progenitor cell survival and growth as they inhibit the *HDAC2*-mediated suppression of IL-6 transcription or upregulate a novel anti-apoptotic long, non-coding RNA (Figure 1) [82,83].

### 5.3. SH2B3/Lnk Mutations Interfere with Key Regeneration Pathways

A key inhibitor of HSC proliferation and inflammation is the lymphocyte adaptor protein Lnk, which is encoded by *SH2B3*, a member of the *SH2B* (Src homology 2-B) adaptor protein family [84]. Aberrant *SH2B3*/Lnk expression is associated with multiple HSC proliferation-related pathologies [85]. In the PERFECT trial, patients with CAD expressed SNP rs3184504 variant of *SH2B3* and exhibited altered *SH2B3* expression levels in the peripheral blood [10,15]. Compared with *SH2B3* knockout mouse models, the downregulated *SH2B3* expression levels promoted CH in various human cells, including myeloid and lymphocytic subsets. In addition to downregulated *SH2B3* expression levels, mutations, such as rs3184504, may enhance inflammation in precursor/immune cells and endothelial cells. The regulatory role of *SH2B3* in hematopoietic and immune regulation, which involves the inhibition of stem cell proliferation (*PLCG1* a.o.) and inflammation by promoting lipid oxidation (*LPCAT2* a.o.), can be classified as a stem cell switch [10,86]. Therefore, *SH2B3*/Lnk mutation-mediated interference of proliferation or inflammatory pathway downregulation may affect PAD/CAD progression based on pleiotropic involvement of the Lnk protein in stem/precursor proliferation, integrin signaling, platelet thrombus formation, and endothelial activation [5,86,87]. Therefore, *SH2B3* is a potential therapeutic target for PAD/CAD.

Thus, recent data indicate that somatic mutations in blood cells significantly increase with age. The mutated blood cells (mainly myeloid cells) secrete increased levels of inflammatory cytokines, such as IL-6, TNFα, and IL-1b, and consequently impair organ function rather than promote tissue regeneration.

## 6. Patient Screening

Recent findings on genetic risk factors that are directly associated with CVD complications (including PAD, CAD, stroke, and hypertension) must be considered, along with comorbid diseases and inflammation, to evaluate the health of individuals with CH, as well as to treat and predict five-year or ten-year mortality. Screening of the population and identification of at least three or more of the following parameters may increase identification of CHIP carriers:

(1) Age: Older adults > 60 years of age [6,28,88].
(2) Blood status: Patients with unexplained anemia or aberrant laboratory parameters (altered myeloid cell/erythroid cell ratio or myeloid/lymphoid ratio) may be associated with an underlying stem cell disorder [89,90].
(3) Pre-existing comorbidities: CAD, PAD, stroke, atherosclerosis, hypertension, diabetes, etc. [5,91].
(4) Inflammatory status: elevated peripheral blood cytokines, such as IL-6, TNF-α, IL-1b, and CRP [78,79,81,91].
(5) Patients with pre-oncology-related factors in the blood may need additional screening for CVD diseases, such as CAD, PAD, and stroke [29].

Thus, experimental and clinical data demonstrate that decreasing or controlling somatic mutation-inducing risk factors, such as inflammation, lipid metabolism, glucose level, diet, and smoking. significantly reduces major adverse cardiovascular events (MACE) and improves stem cell therapy outcomes. State-of-the-art artificial intelligence (AI) algorithms must be utilized to handle these vast amounts of diverse, large medical datasets that include individual genetic features, clinical findings, laboratory biomarkers, and connective computational analyses in stem cell therapy.

## 7. AI-Based R and NR Stratification of Patients with PAD Undergoing Stem Cell Transplantation

AI has contributed to the analysis of clinical data in biomedical fields, including cardiovascular medicine. Additionally, AI assists and supports clinical decisions within a short duration [92].

### 7.1. High Data Quality Is Required for Accurate Prediction Models

The major challenges associated with clinical research on diseases (including PAD) include accessing and retrieving high-quality datasets [15]. The integrated analysis of high-throughput deep-sequencing data, patient phenotypes, images, and SNP loci enables the identification of robust biomarker candidates if controlled, manually curated, and high-quality data are used [93]. Data quality for advanced analysis is critical because it determines the outcomes of subsequent computational analysis [94]. Most studies utilize structured data for predictive modeling and ignore potentially valuable information in unstructured clinical notes, such as doctor reports. This is because of the challenges associated with integrating diverse reports, which are mostly handwritten text documents, into common AI algorithms. The integration of heterogeneous data types across electronic health records (EHRs) through deep learning (DL) techniques is reported to improve the performance of AI prediction models. Zhang et al. demonstrated that the models constructed based on the integration of unstructured clinical notes with structured data outperformed other models that utilize only unstructured notes or structured data [95]. Other DL methods have recently been reviewed for heterogeneous medical data [96] and image analysis [97]. However, AI-assisted clinical decisions should mainly be obtained from structured data to avoid overgeneralization from sparse datasets. Unstructured data allow adequate comprehensibility of the findings. Personalized therapy data, such as individual medical documents obtained from unstructured data, can be integrated. Thus, all data under investigation should be obtained under good practice (GxP) [98]. However, GxP cannot ensure the usage of appropriate or scientifically relevant methods nor the scientific significance of analyses or examinations. The data integrated into an AI study should be carefully analyzed, as using more data may decrease the signal-to-noise ratio [99].

### 7.2. Supporting Decision Making through AI-Based Complex Data Analysis

AI models are currently developed to mitigate the black-box effect, which leads to a lack of interpretability and transparency. Previously, this was the most important reason for the skeptical view of this technology held by patients and clinicians. This is due to the lack of trust in unfamiliar interfaces and hesitancy to rely on a machine or mathematical algorithm for making critical life decisions [100,101].

However, AI-based data analysis algorithms can be an independent extension of previously established statistical approaches for disease risk assessment, such as the recommendations by the American Heart Association/American College of Cardiology (ACC/AHA), which can predict the prognostic risk of CVD based on common risk factors, such as cholesterol, age, smoking, and diabetes [102]. However, several patients are not identified through the classical linear prediction models, and some patients are unnecessarily treated due to false-positive classifications [103,104]. Classical models may thus oversimplify complex, high-dimensional datasets using insufficient parameters. However, the integration of several parameters can result in overfitting the model. This so-called bias-variance dilemma occurs while generating an AI model and has a direct effect on the prediction accuracy, interpretability, and robustness to interpret new data. Complex models with many parameters and a high variance can often lead to overfitting. In these cases, the model adapts itself too closely to the training data and exhibits limited performance on new patients. In contrast, high-biased models that are not complex tend to ignore data points and important features, which ultimately leads to underfitting and decreased model accuracy [105]. This tradeoff must be specifically evaluated and taken into consideration for each dataset and newly trained model by testing different parametrizations. The increased availability of highly efficient AI algorithms has enabled the development of alternative approaches to classical linear prediction models. These models can utilize large, integrative datasets for improved prognosis and diagnosis [94]. In 2014, Dilsizian and Siegel reported that the vast amount of information obtained from patients and pre-clinical studies is too complex and heterogeneous for humans to comprehensively interpret without any technological support [106].

### 7.3. Identification of Responsive Patients for Cell Therapy

The number of patients undergoing genetic testing for various diseases, ranging from cancer to cardiomyopathy, has steadily increased. Additionally, RNA-seq can reveal specific mutational status and utilize the same guidelines used for DNA sequencing [107]. The ethical principles and history underlying clinical genetics will provide clinicians with improved tools to guide their practice and help patients navigate through complex medical-psychosocial terrain [108].

Recent biomedical studies have aimed to identify patient-specific biomarker signatures from high-throughput data to effectively predict postoperative results by stratifying patients into the R and NR groups before therapy. Wolfien et al. proposed a diagnostic strategy to predict the response of patients undergoing coronary artery bypass grafting to BMSC-mediated myocardial repair [10]. Predictive analyses in this case would provide useful insights to identify individuals who are most likely to benefit from BMSC treatment with patient-specific diagnostic characteristics.

This focused panel of molecular targets is consistent with the current comparison of AI approaches and traditional models for using administrative claims with EHR to predict heart-failure outcomes [109]. In this case, approaches with traditional logistic regression were compared in order to predict key outcomes in patients with heart failure, and the added value of predictive models was evaluated using EHR data. In total, 9502 patients (aged ≥ 65 years) with at least one heart-failure diagnosis were identified. Of these, 6113 were included in the training set, while 3389 were used as the testing set. The study comprised a large dataset with standard clinical parameters that do not have increased predictive value for ML-based stratification. The authors initially observed limited predictive capacity in the PERFECT study. Hence, they used additional molecular data, which improved the prediction accuracy of the ML model from 64% to 82% [15]. Further, the authors used specific molecular data, such as RNA-seq data. The stratification accuracy and sensitivity for a larger cohort increased by more than 10% [10]. Firouzi and Sussman suggested that this novel strategic approach of combining transcriptome profiling with precise patient phenotyping and AI-guided feature selection is a potentially valuable tool for advancing personalized medicine and cell-based therapies [110].

### 7.4. A New Diagnostic Tool Supporting Individual Disease Characterization

Generally, all studies utilize different ML methods to identify decision boundaries or specific patterns within patient data to individually characterize patients, identify similar groups or subgroups (referred to as clustering), or assign them to a certain disease, disease stage, or treatment option (referred to as stratification). The underlying “learning step” from data input to prediction involves a test of all available features for their capability of separating patients or subgroups in a supervised or unsupervised manner. The algorithm knows the correct label (e.g., control, treatment, and diagnosis) (the so-called ground-truth) in supervised ML. Each data point consists of manually selected features and the corresponding label (e.g., a data point represents a patient, the features are the clinical data, and the label is the stage of a specific disease). The algorithms attempt to identify a suitable relation between the features and the known label during the learning process, which is usually performed retrospectively on previously generated data. A trained ML model, which serves as a decision boundary, can now be applied to new patient data points to predict a label on previously unlabeled patient data. Here, a well-chosen patient parameter set without high imbalance ratios between the investigated groups and a well-defined ground truth is the key for accurate model performance. However, this can often not be assessed in medical data. New ML-based algorithms for dimensional reduction and visualization, such as t-SNE or UMAP, may enable new possibilities for classifying or reconsidering disease subgroups among patients with unknown labels. These unsupervised technologies for medical cases are predominantly used with heterogeneous data, especially for activated or aberrant signaling pathways (e.g., measured in peripheral blood or single-cell RNA-seq), genetic background (GWAS/TWAS), acquired SNPs, or other comorbidities [111,112]. Based on the integrative data, unique combinations and hybrid forms of diseases will be observed, as has been previously demonstrated on a smaller scale, in which AI will support their identification and characterization [10]. These investigations at this specific individual level would have been impossible without the use of AI. Therefore, these investigations assist in routine clinical decisions, especially concerning personalized cell therapy for PAD.

As previously mentioned, the analysis of medical images is one of the greatest success stories of AI, spanning the analysis of histopathological images [113], electrocardiograms [114], radiographs [115], magnetic resonance imaging slices [116], and many more [108]. Since imaging is very much an emerging field in PAD, this bears high potential because several already-established biomarkers could already be used for an unbiased patient stratification [117]. Similarly, a current review of Flores et al. points towards the infancy of AI in PAD but also foresees a broad spectrum of potential applications [118]. One of the first proofs of concept was implemented by Kim et al. [119] who used a deep convolutional neural network for the detection and assessment of the severity of PAD based on the analysis of brachial and ankle arterial pulse waveforms. These findings were compared with the state-of-the-art ankle-brachial index (ABI) using the virtual patients and showed, according to the authors, that DL may diagnose PAD more accurately and robustly than ABI. This work demonstrates a DL-based arterial pulse waveform analysis for affordable and convenient PAD screening, as well as the open challenges that need to be addressed for real-world clinical applications. However, one current limitation of imaging in PAD is the profound correlation of biomarkers with genetic alterations, which might be addressed with the help of AI, as already demonstrated in other fields, [120] as these ensemble models allow for complex data integration.

Nevertheless, a simple in silico identification of novel subgroups or biomarkers without a specific explanation for the choice a treatment option is insufficient. Therefore, AI algorithms in personalized PAD treatment also must utilize heterogeneous information at the gene level (e.g., GWAS, gene expression, pathway activity, identification of mutations) and phenotype level (e.g., cardiac functionality, angiogenesis potential, and inflammation status) to optimize predictions for cell therapy applications in the future. To achieve this, large patient cohorts are needed to utilize the full potential of personalized medicine therapies assisted by AI approaches.

## 8. Conclusions and Future Perspectives

Cell therapy can improve ABI, T_C_PO_2_, rest pain, pain-free walking distance, ulcer healing, and limb salvage markedly in some patients and moderately in other patients. However, cell therapy outcomes may be improved using state-of-the-art AI-based prediction methods before cell therapy to define the R and NR groups. Previous predictor findings and other studies have reported that up to 40% of CLTI patients are not candidates for revascularization [121]. Hence, autologous personalized cell therapy may be considered as a new standard of care for these patients [10,122]. Growing evidence indicates that genetic screening of patients before stem cell transplantation for ischemic CVD facilitates the assessment of regenerative potential. The average treatment effect of randomized cell therapy trials and precision medicine must be reconsidered based on genetic and clinical laboratory findings by employing an unbiased AI approach to identify the most effective treatment option for patients and decrease treatment expenditure.

Possible next steps should involve larger patient groups and an integration of the AI supportive systems directly in clinical care [123]. This way, all involved stakeholders, including clinicians, patients, and IT practitioners, will become more familiar with the new technologies and the final applications will likewise grow based on the given feedback [124]. However, multimodal data integration, security, federated learning, model performance, understanding, and bias still pose challenges and hurdles to the use of AI in health care in general, which, of course, also applies to PAD [125]. In turn, this is why a mechanistic understanding of genetic alterations and mutational profiles is crucial, in addition to sole AI predictions, as it was recently demonstrated for *SH2B3/Lnk mutations* in mice. A well-suited clinical AI model should not only simply select responding patients but should give indications of the decision being made to get more transparent and accepted.

GWAS and broad cross-sectional studies have demonstrated that race, age, and sex variations must be examined accurately due to the distribution of diseases and genotype variations between ethnic groups, such as Asian, Western, Hispanic, and Afro-American populations. Allogeneic cell and extracellular vesicle (EV) transplantation for CVD is safe and feasible (decreased mutations when transplanting young donor cells/EVs to patients with PAD) and may be optimal for the NR group [57,126]. Taken together, future studies must elucidate strong predictive biomarkers obtained from genotype and phenotype findings of the R and NR groups using ML-based methods to support clinical decisions for managing PAD.

## Figures and Tables

**Table 1:** Positive stem cell-based trial outcomes in patients with CLI.

Author	Year	Cell Type	Target Disease	Route of Tx	*n* =	Outcome	Follow-Up
Tateishi-Yuyama et al. [16]	2002	BMMC and PBMC	CLTI ASO	IM	45	↑ ABI, ↑ TcO_2_, complete ulcer healing; 6/10 (60%) major amputation rates; not shown	4 and 24 weeks
Molavi et al. [17]	2016	BMMC	CLTI ASO	IM rep.	22	↑ SPP, ↑ TcPO_2_, no ABI, reduction in ulcer healing; no adverse effect	24 weeks
Kawamoto et al. [18]	2009	CD34^+^	CLTI TAO	IM	17	↑ TBI, ↑ TcPO2, no ABI, complete ulcer healing; did not affect major amputation rates; 0	3 months
Fujita et al. [19]	2014	CD34^+^	CLTI TAO	IM	11	↑ ABI, ↑ LDP, ↑ SPP, ↑ TcPO2, ↑ pain-free walking distance	12 months

**Table 2:** Negative stem/cell-based trial outcomes in patients with CLTI.

Author	Year	Cell Type	Target Disease	Route of Tx	*n* =	Outcome	Follow-Up
Barc et al. [20]	2006	BMMC ASO	CLTI	IM	29	No differences in ABI and pain; major amputation rates not shown	6 months
Miyamoto et al. [21]	2006	BMMC TAO	CLTI	IM	8	Half of the cell-transplanted patients had long-term adverse events, including death and unfavorable angiogenesis.	24 to 48 months
Benoit et al. [22]	2011	BMMC ASO	CLTI	IM	48	Non-significant difference in amputation rate when compared with placebo	6 months
Teraa et al. [23]	2015	BMMC ASO	CLTI	IA	160	No significant differences were observed for the primary outcome, i.e., major amputation at 6 months, BMMC treated versus placebo groups	6 months
Losordo et al. [24]	2011	CD34 ASO	CLTI	IM	28	Favorable (but non-significant) trend for decreased amputation rate with high-dose administration when compared with placebo	6 months

BMMC, bone marrow-derived mononuclear cell; PBMC, peripheral blood mononuclear cell; ASO, atherosclerosis obliterans; TAO, thromboangiitis obliterans; CLTI, chronic limb-threatening ischemia; Tx, transplantation; IM, intramuscular; IA, intra-arterial; ABI, ankle-brachial index; LDP, laser Doppler perfusion; SPP, skin perfusion pressure.

**Table 3:** Clinical biomarkers of R and NR groups among patients with PAD.

Clinical Trial	Cell Type	Target Disease	Route of Tx	R vs. NR and *p*-Value
Klepanec [25] et al., 2012	BMMC	CLTI	IM and IA	(1) ↑ CD34^+^ cells count in BM (2) ↓ C-reactive protein (3) ↓ serum leucocytes count
Pan [27] et al., 2019	PBMC and CD34^+^	CLTI	IM	(1) Aged ≤ 50 years; *p* < 0.004 (2) Blood fibrinogen > 4 g/L; *p* < 0.003 (3) Arterial occlusion above the knee/elbow; *p* < 0.010 (4) TcPO_2_; *p* < 0.006 (5) Total transplanted CD34^+^ cell count; *p* < 0.046
Madaric [26] et al., 2016	BMMC	CLTI	IM and IA	(1) ↑ CD34^+^ cells count in BM; *p* < 0.001 (2) ↓ C-reactive protein level; *p* < 0.038 (3) ↑ total BMMNCs; *p* < 0.032 (4) Younger patients; *p* < 0.028 (5) ↑ TcPO_2_; *p* < 0.031
Malyar [31] et al., 2014	BMMC	CLTI/PAD	IM and IA	(1) ↓ C-reactive protein level; *p* < 0.01 (2) ↓ interleukin-6; *p* < 0.01
Steinhoff [15] et al., 2017	CD133^+^	CAD/MI	IMyo	(1) ↑ CD34^+^ cell count in PBMC; *p* < 0.027 (2) ↑ CD133^+^ cell count in PBMC; *p* < 0.026 (3) ↑ CD133^+^ and CD117^+^ cell counts in PBMC; *p* < 0.024 (4) ↑ CD146^+^ cell counts in PBMC; *p* < 0.024 (5) EPO in PB; *p* < 0.023
Wolfien [10] et al., 2020	CD133^+^	CAD/MI	IMyo	(1) Machine learning-selected top features of R and NR groups: *PLCG1, LPCAT2, GRB2, AFAP1, AP1B1, KLF8*, *MARK3,* serum *EPO,* serum *VEGF*

R, responder; NR, non-responder; BMMC, bone marrow-derived mononuclear cell; PBMC, peripheral blood mononuclear cell; BM, bone marrow; bFGF, basic fibroblast growth factor; BMMNCs, bone marrow-derived mononuclear cells; EPO, erythropoietin; PB, peripheral blood Tx, transplantation; IM, intramuscular; IA, intra-arterial; CAD, coronary artery disease; PAD, peripheral artery disease.

**Table 4:** Comparative features of TAO and ASO.

TAO	ASO
TAO is an inflammatory vascular disease that predominantly affects small-sized and medium-sized blood vessels of extremities [37].	ASO affects medium-sized or large-sized blood vessels of extremities based on atherosclerotic pathologies [1].
Epidemiology: The prevalence of the PAD varies (0.5 to 5.6% in western Europe, 45 to 63% in India, 16 to 66% in Korea and Japan, and 80% in Israel among Jews of Ashkenazi ancestry) [38].	Epidemiology: The prevalence of ASO was 10.69%, while that of critical limb ischemia was 1.33%. ASO increased by 28.7% in low-income and middle-income countries and by 13.1% in high-income countries [3,39].
Onset: before 45 years [37].	Onset: ASO prevalence and incidence are both age-related and increase by > 10% among patients aged > 60 and 70 years [1].
Risk factors: Tobacco smoking is the major risk factor for the initiation, maintenance, and progression of TAO [38,40,41]	Risk factors: Genetic background, age, cigarette smoking, diabetes, dyslipidemia, and hypertension [1,4,42,43].
In TAO, cellular immunity increased against Collagen type I and III. For example, anti-collagen antibody activity in TAO is higher than that in ASO [44].	
Biomarker: TAO cases exhibit significantly upregulated plasma levels of IL-6, sICAM-1), and sVCAM-1, and the involved arterial tissues express upregulated levels of p-STAT3, ICAM-1, and VCAM-1 [45].	Biomarker: CRP, IL-6, IL-1b, and fibrinogen levels are upregulated in ASO. Macrophages and CRP were detected in atherosclerotic plaque [25,26].

ASO, atherosclerosis obliterans; TAO, thromboangiitis obliterans; PAD, peripheral arterial disease; CRP, C-reactive protein.

**Table 5:** Evidence-based information on peripheral artery disease (PAD) and somatic mutations.

PAD	Share Point	Somatic Mutation
The risk of PAD markedly increases with age. Prevalence of PAD among individuals aged 80–100 years is 22 to 33% [69].	Age	Somatic mutation incidence increases in the aging population by 10 to 20 % at the age of 70 years [6,28].
Atherosclerosis incidence among PAD cases is more than 90% [39].	Atherosclerosis	Presence of somatic mutation in *TET2* increases atherosclerotic lesions of vessels by 3 to 4-fold [5,70].
Diabetic patients have two to four-fold increased risk of developing PAD, CAD, and ischemic stroke [39].	Diabetes mellitus	Clonal mosaic event carriers with type 2 diabetes mellitus were associated with increased (2-fold) prevalence of vascular complications compared to non-carriers with type 2 diabetes mellitus (*p* = 7.7 × 10^−4^) [50,71].
Smoking is the most common risk factor for PAD occurrence, with a population attributable fraction of 44% [1,39].	Tobacco smoking	Tobacco smoking significantly increased the occurrence of somatic mutation to 1000–10,000 mutations per cell [72].

## Data Availability

Not applicable.
